# A Promising Prognostic Indicator for Pleural Mesothelioma: Pan-Immuno-Inflammation Value

**DOI:** 10.3390/jcm14155467

**Published:** 2025-08-04

**Authors:** Serkan Yaşar, Feride Yılmaz, Ömer Denizhan Tatar, Hasan Çağrı Yıldırım, Zafer Arık, Şuayib Yalçın, Mustafa Erman

**Affiliations:** 1 Department of Medical Oncology TR, Hacettepe University Cancer Institute, 06100 Ankara, Turkey; 2 Department of Internal Medicine TR, Sinop Turkeli State Hospital, 57900 Sinop, Turkey; 3 Department of Medical Oncology TR, Nigde Omer Halisdemir Training and Research Hospital, 51100 Nigde, Turkey

**Keywords:** pleural mesothelioma, pan-immune inflammation value, prognostic factors

## Abstract

**Background:** Pleural mesothelioma (PM) is a type of cancer that is difficult to diagnose and treat. Patients may have vastly varying prognoses, and prognostic factors may help guide the clinical approach. As a recently identified biomarker, the pan-Immune-Inflammation-Value (PIV) is a simple, comprehensive, and peripheral blood cell-based biomarker. **Methods:** The present study represents a retrospective observational analysis carried out within a single-center setting. Ninety-five patients with PM stages I–IV were enrolled in the study. We analyzed the correlation between patients’ demographic characteristics, clinicopathological factors such as histological subtypes, surgery status, tumor thickness, blood-based parameters, and treatment options with their prognoses. PIV was calculated by the following formula: (neutrophil count × monocyte count × platelet count)/lymphocyte count. Additionally, blood-based parameters were used to calculate the platelet-to-lymphocyte ratio (PLR), neutrophil-to-lymphocyte ratio (NLR), and systemic immune inflammation index (SII). **Results**: We categorized the patients into two groups, low PIV group (PIV ≤ 732.3) and high PIV group (PIV > 732.3) according to the determined cut-off value, which was defined as the median. It was revealed that high PIV was associated with poor survival outcomes. The median follow-up period was 15.8 months (interquartile range, IQR, 7.1 to 29.8 months). The median overall survival (OS) was significantly longer in patients in the low PIV group (median 29.8 months, 95% confidence interval (CI), 15.6 to 44) than the high PIV group (median 14.7 months, 95% CI, 10.8 to 18.6 *p* < 0.001). Furthermore, the study revealed that patients with low PIV, NLR, and SII values were more likely to be eligible for surgery and were diagnosed at earlier stages. Additionally, these markers were identified as potential predictors of disease-free survival (DFS) in the surgical cohort and of treatment response across the entire patient population. **Conclusions**: In addition to well-established clinical factors such as stage, histologic subtype, resectability, and Eastern Cooperative Oncology Group (ECOG) performance status (PS), PIV emerged as an independent and significant prognostic factor of overall survival (OS) in patients with PM. Moreover, PIV also demonstrated a remarkable independent prognostic value for disease-free survival (DFS) in this patient population. Additionally, some clues are provided for conditions such as treatment responses, staging, and suitability for surgery. As such, in this cohort, it has outperformed the other blood-based markers based on our findings. Given its ease of calculation and cost-effectiveness, PIV represents a promising and practical prognostic tool in the clinical management of pleural mesothelioma. It can be easily calculated using routinely available laboratory parameters for every cancer patient, requiring no additional cost or complex procedures, thus facilitating its integration into everyday clinical practice.

## 1. Introduction

Mesothelioma is a rare cancer that predominantly affects the pleural cavity and is most commonly caused by exposure to asbestos [1]. Symptoms and signs may be non-specific, leading to difficulties in diagnosis and anatomic localization usually renders curative treatment impossible. The median overall survival (OS) for localized disease is 12–30 months and 8–14 months for advanced disease [2]. The demographic characteristics of mesothelioma caused by environmental exposure are somewhat different from those that occur industrially. The situation in Turkey is unique in the world, with exceptionally high levels of environmental exposure. Environmental asbestos exposure usually starts right after birth, so the median age of mesothelioma diagnosis is lower than that caused by industrial exposure. Some studies conducted in our country have shown that the median age is below 60, especially in areas where environmental exposure is high [3].

Malignant pleural mesothelioma presents with different histologic subtypes, each associated with distinct incidences and prognoses. According to Janes et al., the epithelioid subtype accounts for 50–60% of cases and is associated with a more favorable prognosis. In contrast, the sarcomatoid subtype, observed in approximately 10% of patients, shows poor prognosis and resistance to therapy, whereas the biphasic subtype (30–40%) displays mixed features. Less common variants include desmoplastic and unclassified subtypes [4].

Various treatment modalities are currently available, including chemotherapy, immunotherapy, and surgery, all of which are components of multimodal therapy. However, the role of surgery and surgical approaches remain matters of ongoing discussion. For many years, patients with malignant pleural mesothelioma had limited systemic treatment options with proven efficacy, and therapeutic approaches were largely palliative. The EMPHACIS trial was the first to demonstrate that systemic therapy could improve survival and provide symptom control in this population. In this phase III randomized study, the combination of cisplatin and pemetrexed significantly improved median overall survival compared to cisplatin alone (12.1 months vs. 9.3 months; *p* = 0.02), with higher objective response rates (41% vs. 17%) and better symptom management. Based on these findings, cisplatin–pemetrexed became the standard first-line treatment for patients with unresectable epithelioid mesothelioma and has been widely adopted in international clinical guidelines [5]. Subsequently, more promising results were achieved through studies focusing on immunotherapy. In a trial comparing the combination of nivolumab and ipilimumab with platinum-based chemotherapy in previously untreated, unresectable malignant pleural mesothelioma patients, a significant improvement in overall survival was demonstrated. Based on these findings, the dual immunotherapy regimen has become a leading first-line treatment option [6]. In addition, radiotherapy, an important part of treatment, is administered in palliative, adjuvant, or neoadjuvant settings. The radiotherapy technique and delivered doses vary depending on disease extent and the chosen surgical approach. Several studies provide evidence, particularly for delaying local recurrence [7]. The role of surgery as part of multimodal treatment remains controversial in the literature, with conflicting outcomes reported. In prospective randomized trials such as MARS 1 and MARS 2, surgical intervention did not demonstrate a survival benefit and, in some cases, was associated with detrimental effects. However, in contrast to these findings, real-world data exist suggesting a survival advantage with surgical treatment [8,9,10].

Although several biomarkers have been studied for mesothelioma such as mesothelin, fibulin-3, microRNAs (miRNAs), soluble mesothelin-related peptides (SMRPs), and high mobility group box 1 (HMGB1), only a few have shown consistent clinical relevance. Among these, loss of BAP1 protein expression has gained attention as a promising example of a validated molecular biomarker associated with both diagnosis and survival. Notably, a recent multicenter validation study demonstrated that BAP1 loss detected by immunohistochemistry predicts improved overall survival in patients treated with first-line platinum and pemetrexed chemotherapy, further supporting its potential clinical utility as a prognostic and predictive biomarker in pleural mesothelioma [11,12].

It is well known that inflammation has an effect on many phases of cancer development, metastasis, and resistance to chemotherapy [13,14]. Each blood cell performs its own function and reflects systemic and tumor immunity by working together. Neutrophils release pro-tumor cytokines, chemokines, and reactive oxygen species and may promote immunosuppression, thus changing the tumor microenvironment. However, tumor-associated macrophages (TAMs) have the characteristic of plasticity and can acquire various phenotypes and affect many steps of carcinogenesis [15]. Tumor-associated macrophages (TAMs), which originate from monocytes or tissue-resident precursors, display the ability to transition between pro-inflammatory M1 and immunosuppressive M2 phenotypes influenced by cytokines such as IFN-γ, IL-4, and IL-10. Within the tumor microenvironment, TAMs facilitate tumor progression by stimulating angiogenesis, remodeling the extracellular matrix, and suppressing immune responses through factors including VEGF, MMPs, PD-L1, and IL-10 [16]. Moreover, platelets have one of the largest reservoirs of angiogenic and mitogenic factors that actively participate in the metastatic cascade. They protect tumor cells from immune surveillance, modulate tumor cell invasion, and promote angiogenesis [17]. Similar to TAMs, T lymphocytes may affect and accelerate tumor development with cytokines and chemokines [18]. An elevated monocyte count is associated with poor prognosis, and monocytes are another type of blood cell that accelerates angiogenesis and tumor growth by the secretion of tumor necrosis factor alpha and VEGF. In the tumor microenvironment, monocytes can differentiate into tumor-associated macrophages [19], as discussed above. All these effects emphasize the importance of monocytes in cancer prognosis, just like other blood-based cells.

Based on these findings, recently, researchers have focused on predicting the prognosis of various tumors using parameters such as NLR, SII, and PLR [20].

In more recent times, there has been considerable interest in a systemic inflammation indicator known as the PIV, derived from a comprehensive analysis of blood-based parameters including neutrophil, platelet, monocyte, and lymphocyte counts. This value is considered a strong candidate for predicting survival across various solid tumor types. Sato S et al. have recently shown that PIV can be a good and useful predictive marker in determining prognosis in patients with stage I-III colorectal cancer undergoing surgery [21]. Similarly, Şahin et al. showed that a low PIV in patients with breast cancer may independently predict survival and a good response to chemotherapy in patients treated with neoadjuvant chemotherapy [22]. We evaluated the prognostic value of PIV in pleural mesothelioma (PM) patients in our study.

## 2. Materials and Methods

### 2.1. Patient Population and Data Collection

The electronic records of patients over 18 years old admitted to Hacettepe University Cancer Center between 01/2014 and 01/2021 with histologically or cytologically confirmed PM were reviewed. Basal characteristics (ECOG PS, gender, age, histological type, surgery status, tumor thickness), treatment modalities, inflammatory blood-based parameters, and calculated PIV were recorded as well as the survival data.

Pretreatment PLR, SII, NLR, and PIV were computed based on absolute blood parameters, including lymphocyte, neutrophil, monocyte, and platelet counts, and their outcomes were documented. PIV was calculated with the following equation: (neutrophil count × platelet count × monocyte count)/lymphocyte count [23].

We calculated NLR by dividing the number of neutrophils by the number of lymphocytes, and likewise computed PLR (platelet-to-lymphocyte ratio). Then, we determined SII using the formula: (neutrophil count × platelet count) divided by the lymphocyte count.

The median values of NLR, SII, PLR, and PIV were identified as the cut-off values. Afterward, patients were categorized into two groups: patients below the median were considered as the low-value group, and patients above the median were considered as the high-value group.

Histologically, patients were evaluated in two main categories: epithelial (68.4%) and non-epithelial (31.6%) (biphasic, sarcomatoid, and desmoplastic, not otherwise specified). We used the TNM 8 staging system, and patients were classified as having one of four stages: 25 in stage I (26.3%), 17 in stage II (17.9%), 36 in stage III (37.9%), and 17 in stage IV (17.9%).

Recently, some researchers have investigated tumor thickness and prognosis. Therefore, we wanted to include high and low tumor thickness in our analyses according to the cut-off we determined. We showed the effect of tumor thickness on survival in our analyses. Tumor thickness was measured according to the International Association for the Study of Lung Cancer (IASLC) in their mesothelioma staging project, and the median value was determined and accepted as the cut off (median thickness is 17 mm). The study group divided the patients into groups according to the cut-offs they determined for sum of maximum pleural thickness (Psum) in terms of prognosis and worked to evaluate the prognosis in more detail. Psum ≤ 12, >12 mm, ≤ 30 mm, and > 30 mm. In our study, among the 73 patients for whom the Psum could be evaluated, 23 (31.5%) had a Psum < 12 mm, 28 (38.4%) had a Psum between >12 mm and ≤30 mm, and 22 (30.1%) had a Psum > 30 mm [24].

We also created more specific subgroups to evaluate various patient characteristics: the ‘surgery group’, which included patients who underwent surgery at stages I–III, and the ‘non-surgery group’, consisting of medically inoperable locally advanced patients (stages I–III) and stage IV patients.

*In the surgical population*, there were 29 patients and multimodal treatment was applied; all patients received adjuvant platinum-based chemotherapy and, if appropriate, radiotherapy. For resectable patients, surgical procedures such as pleural decortication (P/D) and extrapleural pneumonectomy (EPP) were applied. In total, 6 patients underwent extrapleural pneumonectomy and 23 patients underwent pleural decortication. Surgery was performed on patients with epithelioid histology, ECOG PS 0–1, disease limited to a single hemithorax, and no serious comorbidities. Patients who were predicted to undergo macroscopic complete resection were selected. We preferred P/D for frail group and for patients who we predicted would have more postoperative complications. EPP was planned for fit patients with expected better tumor response. In total, 6 patients who underwent EPP received 50 Gy radiotherapy, and *intensity-modulated radiation therapy* (IMRT) was applied to 14 patients who underwent P/D (25–30 Gy) (it was not applied to 9 patients because it was not suitable).

### 2.2. Statistics

The overall survival was defined as the duration from the diagnosis until the final follow-up assessment and/or occurrence of death. Survival was estimated according to the Kaplan–Meier method. We used the log-rank test to compare the survival of subgroups and Cox proportional hazards model for hazard ratio (HR) and corresponding 95% confidence interval (CI). Variables that were significantly associated with OS in univariate Cox regression analysis were evaluated with multivariate modeling. All statistical tests were two-tailed, and a *p*-value < 0.05 was considered statistically significant. The statistical analysis was conducted using SPSS, version 25.0 (IBM Inc., Armonk, NY, USA).

## 3. Results

Ninety-five patients were analyzed in our study. Of the patients, 63.2% were male and 36.8% were female. Of the patients, 55.8% were under the age of 65, and the median age was 62.2 years (range: min:32–max: 85) (Table 1). The majority of retrospectively reviewed patients had an ECOG performance status of 0–1 (84.2%). All patients were included in the analysis; however, only two patients received best supportive care without systemic anticancer treatment.

Cut-off value for PIV, NLR, SII, and PLR was determined as the medians. The cut-off values for PIV/SII/NLR/PLR were 732.3, 1165, 3.8, and 210, respectively.

**Figure 1:**
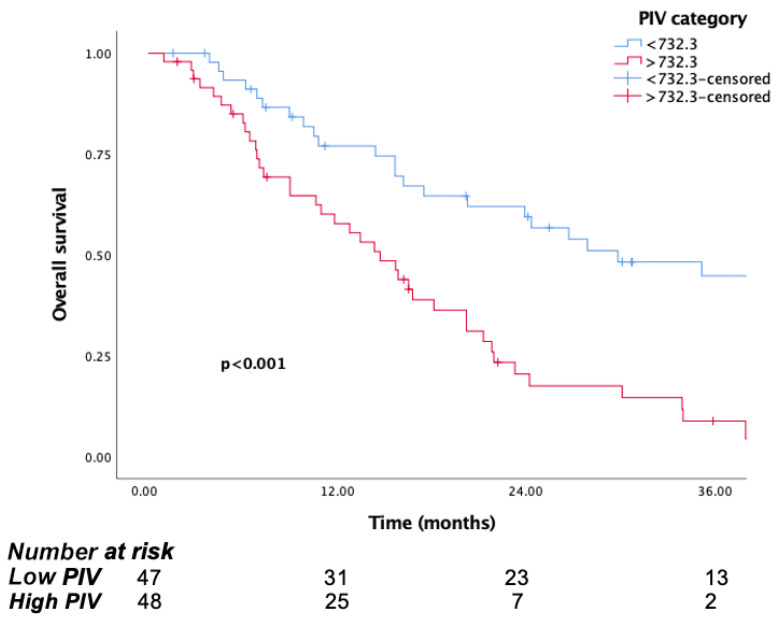
Kaplan–Meier curves for overall survival (OS) in patients with malignant pleural mesothelioma according to baseline PIV.

**Figure 2:**
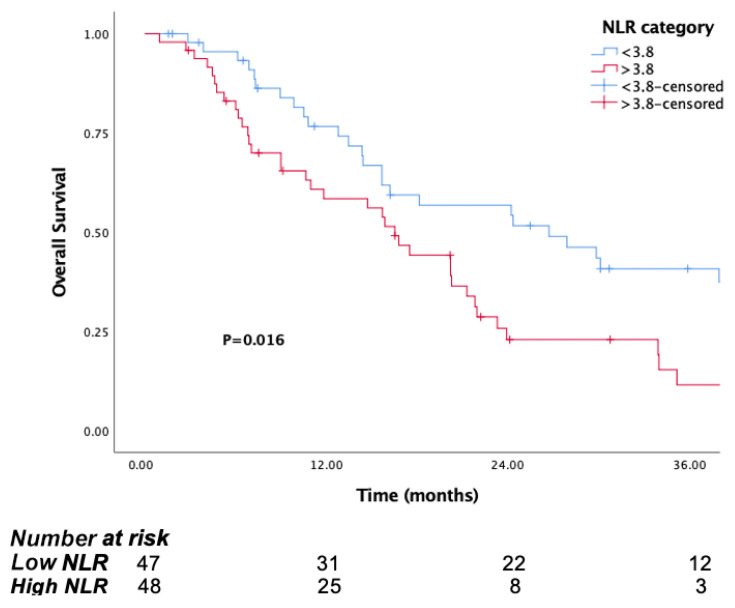
Kaplan–Meier curves for overall survival (OS) in patients with malignant pleural mesothelioma according to baseline NLR.

**Figure 3:**
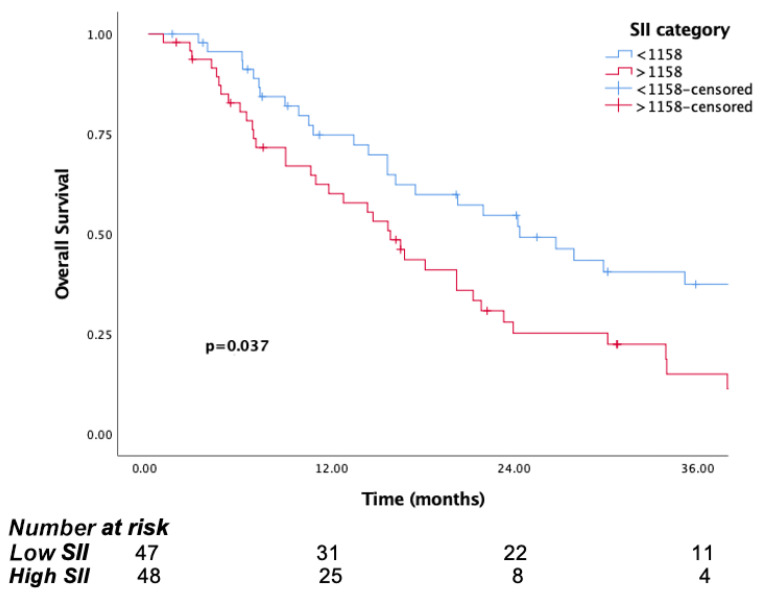
Kaplan–Meier curves for overall survival (OS) in patients with malignant pleural mesothelioma according to baseline SII.

**Figure 4:**
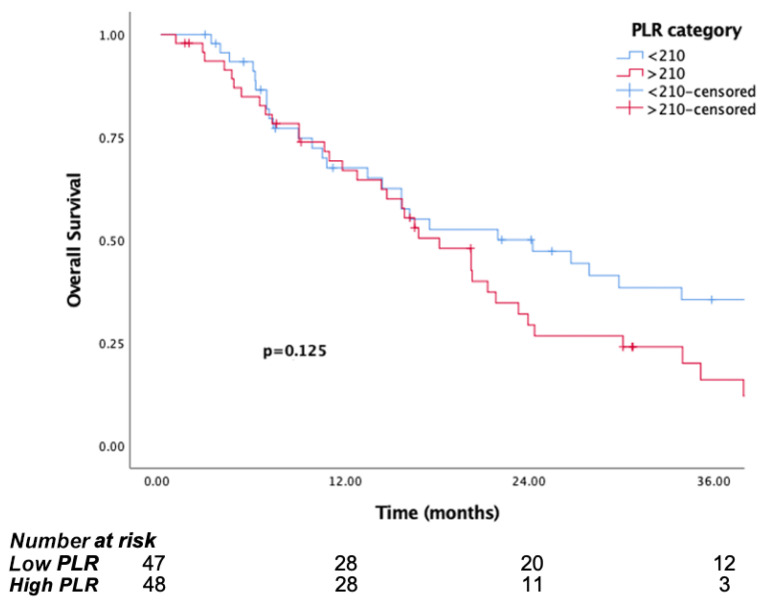
Kaplan–Meier curves for overall survival (OS) in patients with malignant pleural mesothelioma according to baseline PLR.

The median follow-up time was 15.8 months (IQR, 7.1 to 29.8). The median OS was significantly longer in patients in the low PIV group (PIV ≤ 732.3) (29.8 months, 95% CI, 15.6–44) than the high PIV group (PIV > 732.3) (14.7 months, 95% CI: 10.8–18.6, *p* <0.001) (Figure 1). Likewise, lower NLR and SII were associated with longer survival (*p* = 0.016, *p* = 0.037, respectively) (Figure 2 and Figure 3). But there was no statistical significance between low PLR for OS (*p* = 0.125) (Figure 4).

In addition to the blood parameters, there was a significant relationship between ECOG PS (ECOG = 0–1 vs. ECOG ≥ 2), histological type, tumor thickness, and surgery status on OS, but there was no significant relationship between age and gender in survival analysis. As with many solid tumors, one of the most important prognostic factors was the stage, and the Kaplan–Meier analysis showed that survival decreased from stage I to IV, as expected. In the survival analysis, the median OS for stage I was 42.4 months (95% CI: 25.3–59.5), while it was 11.8 months (95% CI: 0–23.9) for stage IV, and these findings were also statistically significant (*p* < 0.001).

Additionally, in our study, we sought to answer the question of whether the inflammation parameters of the patients indicate their suitability for surgery or if higher values tend to correlate with a more advanced stage.

When stage IV patients were excluded, 77.5% of the patients in the group with high PIVs were not suitable for surgery, while this rate was 55.3% in the group with low PIVs. In total, 73% (36 of 49 patients) in the high NLR group and 75% (36 of 48 patients) in the SII group were found to be unsuitable for surgery. This rate was lower in the low group. However, there was no difference between the high and low PLR groups. Additionally, the high PIV group tended to have more advanced stage or extensive disease. While 66.7% of the patients were in stage III–IV, 33.3% were in stage I–II in the high PIV group. In contrast, 44% of the patients were in stage 3–4 at the time of diagnosis in the low PIV group. All these results showed that high PIV, NLR, and SII provided us with evidence not only about prognosis but also about the patient’s stage at diagnosis and suitability for surgery.

**Figure 5:**
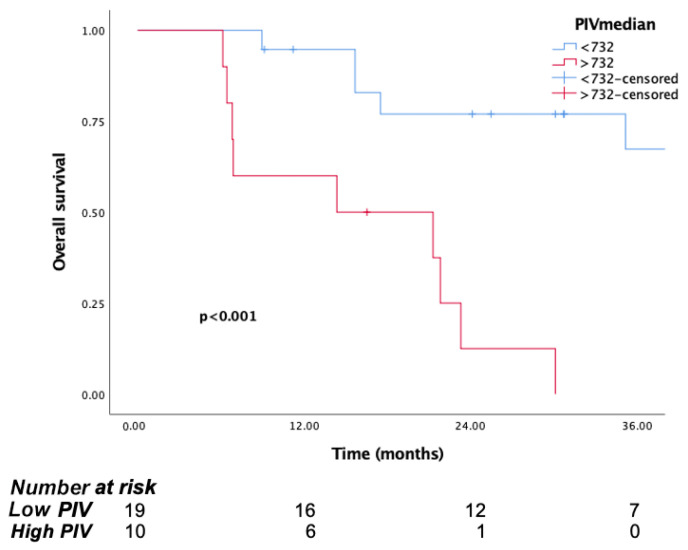
Kaplan–Meier curves for overall survival (OS) in patients with malignant pleural mesothelioma according to baseline PIV, for surgical group.

*In the surgical group*, mOS was 35.1 months (95% CI: 12.9–57.3), whereas for the non-surgical group, it was 15.8 months (95% CI: 12.4–19.2). It should be noted that if patients are evaluated by an experienced surgeon and deemed suitable for surgery, survival significantly increases. We also evaluated the association between surgery and PIV. According to the PIV, the mOS was not reached in the low PIV group, whereas it was 14.3 months (95% CI: 0.9–34.1) in the high PIV group (*p* < 0.001). Consistently, the 1- and 3-year survival rates in the low PIV group were 77% and 67%, respectively, in the high PIV group, the 1-year survival rate was 60%, and no patient survived more than 3 years (Figure 5). These results suggest that patients with lower PIVs before surgery have better survival rates. Although the number of patients is limited, PIV can be a guiding biomarker for the evaluation of surgery group.

All patients who underwent surgery received adjuvant platinum-based therapy (cisplatin/carboplatin-pemetrexed). Twenty patients received trimodal treatment; that is, they received adjuvant radiotherapy and chemotherapy after surgery.

**Figure 6:**
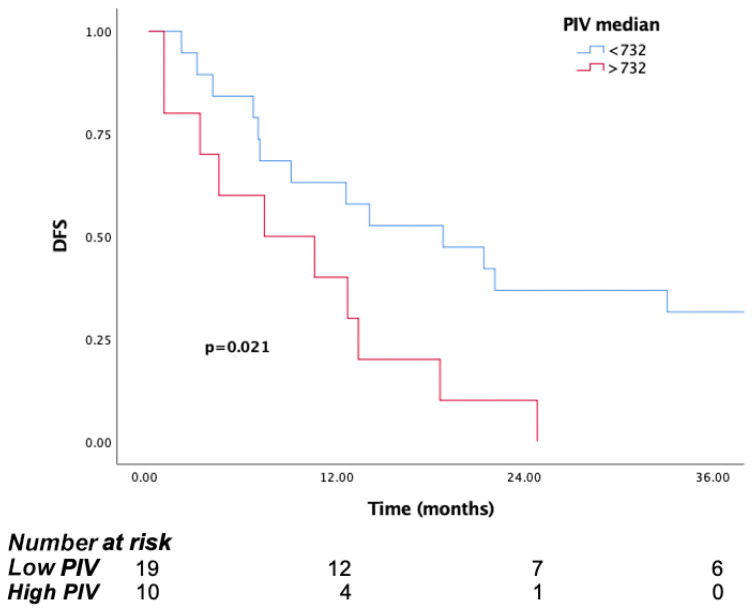
Kaplan–Meier curves for disease-free survival (DFS) in patients in surgical group according to baseline PIV.

The treatment effectiveness of the patients after adjuvant treatment was evaluated. The median disease-free survival (DFS) was 12.6 (95% CI: 7.02–18.1). mDFS was 18.8 months (95% CI: 6.2–31.3) in the low PIV group and 7.4 months (95% CI: 0–16.8) in the high PIV group, with a statistically significant difference (*p* = 0.021) (Figure 6).

*In the non-surgical group* (stage I–III surgery ineligible and stage IV), mOS was 15.8 months (95% CI: 12.4–19.2). The efficacy of the first-line treatment in patients from the non-surgical group was assessed, revealing a median progression-free survival (PFS) of 6 months (95% CI: 3.88–8.11).

Among these patients, 42 received cisplatin-pemetrexed, 11 received carboplatin-pemetrexed, 7 received single-agent pemetrexed, and 4 received other agents (single-agent pemetrexed, gemcitabine, vinorelbine). Additionally, two patients could not receive treatment due to poor performance. Within this group, 30% of the patients received second- and third-line treatments after disease progression.

**Figure 7:**
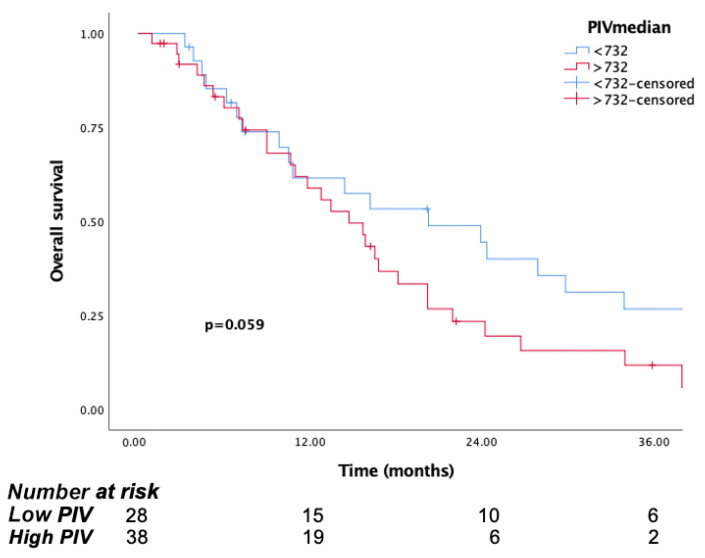
Kaplan–Meier curves for overall survival (OS) in patients with non-surgical group according to baseline PIV.

In this cohort, the mOS was 20.2 months (95% CI: 5.8–34.6) in the low PIV group, compared to 14.7 months (95% CI: 10.4–18.9) in the high PIV group (*p* = 0.059). In line with these findings, the 1-year and 3-year survival rates were 61.5% and 26% in the low PIV group, respectively, while in the high PIV group, they were 58% and 11%, respectively (Figure 7).

Univariate analysis showed that pretreatment worse ECOG, performance status, high PIV, high NLR, high SII, non-surgical group, non-epithelioid type, and high tumor thickness were associated with poorer OS. When multivariate analysis was performed with these parameters, high PIV (HR = 3.35, 95% CI: 1.29–8.76, *p* = 0.013) and non-epitheloid type (HR = 2.46, 95% CI: 1.2–5.05, *p* = 0.013) were independently associated with poor survival outcomes (Table 2).

## 4. Discussion

The impact of inflammation on the onset and progression of cancer is now clearly established, and blood cells are important mediators of this impact [25]. Recently, many studies have been carried out with various combined indicators such as NLR, SII, and PLR, and their effects on predicting the prognosis of some cancers have been established. Al Jarroudi O et al. studied the predictive properties of NLR and PLR in patients with inflammatory breast cancer. Patients with high NLR and PLR had worse outcomes in terms of both PFS and OS in this study [26].

In another study, Lou C et al. investigated the correlation between NLR, PLR, and HALP (hemoglobin and albumin levels and lymphocyte and platelet counts) for neoadjuvant therapy and prognosis for triple-negative breast cancer. The study similarly demonstrated poor outcomes for PLR and NLR [27].

Another combined score, SII, was recently studied by Huang Y et al. It has been shown that it may be an important prognostic factor in postoperative survival in patients with endometrial cancer [28].

Based on previous studies, we thought that PIV could be more guiding and more comprehensive in prognosis, because it uses monocytes as an additional component of the immune system. The first clinical studies on the subject were recently performed by Fuca G et al. The first trial demonstrated that patients with metastatic melanoma with high PIV are associated with poor survival rates and also associated with poor treatment response to targeted therapies (BRAF/MEK inhibitors) and immunotherapies. In another study, Fuca G et al. similarly demonstrated the prognostic importance of PIV in patients with metastatic colorectal cancer [29,30].

While studies have been conducted on many types of cancer using blood-based parameters, and no biomarkers have yet been identified that are simple, practical, and applicable in daily practice for PM, researchers have begun to study these predictors in PM. Yeap BY et al. obtained some evidence about prognosis in their study in patients with malign PM, including subtypes, tumor burden, and NLR. They emphasized the connection between NLR and tumor burden with prognosis [31]. Kao SC et al. revealed that low calreticulin and high NLR may predict poor prognosis in patients who underwent extrapleural pneumonectomy [32]. In another study conducted by Karakaya et al., it was shown that the SII may be an important parameter for the course of prognosis in both malignant pleural and peritoneal mesothelioma [33]. Similarly, a study including 97 patients showed that SII is a strong, non-invasive prognostic marker. High SII has been associated with poor prognosis [34].

However, better and more comprehensive models have also been considered. A study by Pinato DJ et al. included more variables and found that, in addition to NLR and PLR, high CRP value and low albumin levels were also associated with poor survival [35].

In another recent study closely paralleling our work, the relationship between PIV and malignant mesothelioma was evaluated. This study demonstrated that high pre-treatment PIV was significantly associated with poor outcomes (HR: 2.01, 95% CI: 1.32–4.79, *p* = 0.03). Similarly, other inflammatory parameters such as high SIRI and NLR were also linked to worse survival outcomes. In contrast, indices like the advanced lung cancer inflammation index (ALI) and HALP showed associations with better prognoses [36].

Our analyses supported these findings in the literature. We showed that NLR and SII are related to prognosis, but PLR is insufficient to indicate prognosis

Our data have revealed that PIV is a new independent prognostic factor for PM, in addition to the other better-known factors like stage, ECOG PS, surgery, and histological subtype. It was observed that PIV not only predicted prognosis but also provided clues for determining treatment strategies. It has been shown that it can guide the application of more aggressive treatment strategies, such as surgery, at an earlier stage. Two major studies that evaluated the role of surgery in malignant pleural mesothelioma are MARS 1 and MARS 2. In the MARS 1 trial, patients underwent extrapleural pneumonectomy (EPP) followed by radiotherapy in the surgical arm, while no surgery was performed in the control arm; both groups subsequently continued with standard treatments. In MARS 2, patients were randomized to undergo pleurectomy/decortication followed by chemotherapy or to receive chemotherapy alone. In both studies, platinum-based chemotherapy was administered prior to randomization. Neither study demonstrated a clear survival benefit associated with surgery [8,9]. In addition, in the study conducted by Nakamura et al., patients were divided into two groups: those who underwent curative surgery following three cycles of neoadjuvant platinum-based chemotherapy, and those who refused surgery. The study found that overall survival was significantly lower in the non-surgical group [10]. Given the conflicting evidence in the literature, we analyzed the surgical group separately in our study and proposed that the evaluated parameters could help guide patient selection.

Consistent with the literature, our study showed that the patient’s life expectancy is prolonged if the patient is suitable for surgery and a good surgery can be performed.

Other blood-based parameters like NLR, SII, and PLR were also found to be statistically significant by univariate analyses, but they were not found to be independent factors by multivariate analyses. As such, PIV has outperformed other blood-based parameters. It can be easily calculated, using values that are readily available for every cancer patient, and requires absolutely no extra cost. There are some limitations in our study. This study was conducted in a single center, the sample size is limited, and the follow-up duration is relatively short. Yet, it must be noted that PM is not a common cancer, and most studies do not have larger sample sizes. Furthermore, no validation with an external group could be carried out to confirm our results. We have not evaluated the effect of PIV on treatment outcomes, so it cannot be used as a predictive marker.

Despite these limitations, we have shown that a simple value calculated by blood parameter pre-treatment can be used to predict prognosis in patients with malignant pleural mesothelioma.

## 5. Conclusions

In summary, PIV is a simple, inexpensive, comprehensive and non-invasive biomarker that can help better predict the outcome of PM patients. If further studies support its role, it can be a useful tool for assessing prognosis.

## Figures and Tables

**Table 1:** Demographic and clinical characteristics of the study population.

Characteristic	*n* (%)
Age, years	
Median (range)	62 (32–85)
<65	53 (55.8)
≥65	42 (44.2)
Gender	
Female	35 (36.8)
Male	60 (63.2)
Histological type	
Epiteloid	65 (68.4)
Non-epiteloid	30 (31.6)
Stage	
I	25 (26.3)
II	17 (17.9)
III	36 (37.9)
IV	17 (17.9)
Chemotherapy regimen	
Cisplatin pemetrexed	66 (69.5)
Carboplatin pemetrexed	16 (16.8)
Pemetrexed	7 (7.4)
Other agents	4 (4.2)
Ineligible for treatment	2 (2.1)
Pretreatment ECOG	
0–1	80 (84.2)
≥2	15 (15.8)
Tumor thickness	
Median (IQR)	17 mm (12–35)
Surgical procedures	
Pleural decortication (P/D)	23 (79)
Extrapleural pneumonectomy (EPP)	6 (20)
PIV	
Median (range)	732.3 (113.6–10612.1)
NLR	
Median (range)	3.8 (0.85–18)
PLR	
Median (range)	210 (38.8–822.5)
SII	
Median (range)	1165 (162.3–7320.4)

**Table 2:** Univariate and multivariate analyses for progression-free and overall survival.

Variable	Univariable Analysis			Multivariable Analysis		
	HR	95% CI	*p* Value	HR	95% CI	*p* Value
Age (years) <65 vs. ≥65	1.23	0.76–2.0	0.399			
Gender Male vs. Female	1.16	0.69–1.97	0.564			
Pretreatment Ecog 0–1/2–4	2.01	1.1–3.98	0.023			
PIV High vs. Low	3.04	1.78–5.20	<0.001	3.35	1.29–8.76	0.013
NLR High vs. Low	1.83	1.12–2.99	0.016			
PLR High vs. Low	1.46	0.89–2.40	0.125			
SII High vs. Low	1.67	1.03–2.73	0.037			
Histological type epithelioid/non-epithelioid	1.79	1.09–2.95	0.025	2.46	1.20–5.05	0.013
Surgery (stage I–III) Non-surgery (ineligible for surgery stage I–III and stage IV)	0.38	0.21–0.68	<0.001			
Tumor thickness High vs. Low	2.53	1.39–4.58	0.002			

## Data Availability

The data that support the findings of this study are available from the corresponding author upon reasonable request.

## Abbreviations

The following abbreviations are used in this manuscript:

PM	Pleural mesothelioma
PIV	Pan-Immune-Inflammation-Value
PLR	Platelet-to-lymphocyte ratio
NLR	Neutrophil-to-lymphocyte ratio
SII	Systemic immune inflammation index
DFS	Disease-free survival
ECOG PS	Eastern Cooperative Oncology Group Performance Status
TAMs	Tumor-associated macrophages
IASLC	International Association for the Study of Lung Cancer
Psum	Sum of maximum pleural thickness
P/D	Pleural decortication
EPP	Extrapleural pneumonectomy
IMRT	Intensity-modulated radiation therapy
HALP	Hemoglobin and albumin levels and lymphocyte and platelet counts
